# Quadriceps Strength, Postural Stability, and Pain Mediation in Bilateral Knee Osteoarthritis: A Comparative Analysis with Healthy Controls

**DOI:** 10.3390/diagnostics13193110

**Published:** 2023-10-01

**Authors:** Mastour Saeed Alshahrani, Ravi Shankar Reddy

**Affiliations:** Department of Medical Rehabilitation Sciences, College of Applied Medical Sciences, King Khalid University, Abha 61421, Saudi Arabia; msdalshahrani@kku.edu.sa

**Keywords:** knee osteoarthritis, quadriceps strength, postural stability, pain mediation, musculoskeletal health, functional impairment

## Abstract

Bilateral knee osteoarthritis (OA) poses significant challenges to individuals’ functional abilities, including quadriceps strength, postural stability, and pain perception. Understanding the complex relationships among these factors is crucial for enhancing knee OA management strategies. The primary objective of this research is to evaluate and draw comparisons between the strength of the quadriceps and the level of postural stability in two distinct groups: individuals afflicted with bilateral knee OA and those who are healthy. Furthermore, the study seeks to examine the potential correlation between the strength of the quadriceps and the level of postural stability in individuals with knee OA. In addition to this, an investigation into the potential mediating effect of pain on the relationship between these physiological factors will also be conducted. A total of 95 participants with bilateral knee OA and 95 healthy controls were recruited. Quadriceps strength was assessed using dynamometry and postural stability was evaluated through anterior–posterior and medial–lateral sway measurements along with the ellipse area using a force plate. Pain levels were measured using the Visual Analog Scale (VAS). Mediation analysis was employed to explore the role of pain in mediating the relationship between quadriceps strength and postural stability. Statistical analyses included t-tests, Pearson correlation coefficients, and mediation analysis. Knee OA participants exhibited significantly lower quadriceps strength (1.08 Nm/kg ± 0.54) compared to controls (1.54 Nm/kg ± 0.57, *p* < 0.001). They also demonstrated compromised postural stability with increased anterior–posterior sway (9.86 mm ± 3.017 vs. 2.98 mm ± 1.12, *p* < 0.001), medial–lateral sway (7.87 mm ± 2.23 vs. 3.12 mm ± 1.34, *p* < 0.001), and larger ellipse area (935.75 mm^2^ ± 172.56 vs. 436.19 mm^2^ ± 135.48, *p* < 0.001). Negative correlations were observed between quadriceps strength and postural stability variables (r = from −0.43 to −0.51, *p* < 0.001). Pain significantly mediated the relationship between quadriceps strength and postural stability variables (*p* < 0.05). This study highlights the associations between quadriceps strength, postural stability, and pain mediation in individuals with bilateral knee OA. Our findings emphasize the need for targeted interventions addressing quadriceps weakness and compromised postural stability. Additionally, the mediation effect of pain underscores the complexity of these relationships, offering insights for more effective management strategies.

## 1. Introduction

Osteoarthritis (OA) is a prevalent degenerative joint disease affecting millions of individuals worldwide, and it represents a significant burden on healthcare systems and patient quality of life [1]. Among various forms of OA, knee OA is ubiquitous and debilitating, impacting functional mobility and postural stability [2]. One of the key factors influencing knee OA progression and management is quadriceps muscle strength, which plays a crucial role in maintaining joint stability and overall lower limb function [3]. In addition to quadriceps strength, postural stability is another essential aspect of knee OA, as it affects the ability to maintain balance and prevent falls, a major concern for individuals with the condition [4]. Furthermore, pain, a prominent symptom in knee OA, can exacerbate functional limitations and influence the relationship between quadriceps strength and postural stability [5].

Compared to healthy controls, the proposed study aims to comprehensively investigate the interplay between quadriceps strength, postural stability, and pain mediation in individuals with bilateral knee OA. Understanding these relationships can shed light on the complex mechanisms involved in knee OA and contribute to the development of targeted interventions for improving functional outcomes and reducing the burden of the disease [6,7]. The first objective of this study is to assess and compare quadriceps strength and postural stability between individuals with bilateral knee OA and healthy controls. Previous research shows that knee OA leads to quadriceps muscle atrophy due to factors such as pain, inflammation, and disuse [8]. By evaluating quadriceps strength in both groups, we aim to quantify the extent of weakness in the knee OA cohort and identify potential targets for interventions to improve muscle function and joint stability [9]. Conversely, postural stability refers to the ability to maintain an upright position while standing or performing dynamic movements [10]. It is influenced by various factors, including sensory inputs, muscle strength, and joint integrity. In knee OA, postural stability is compromised due to pain, joint damage, and muscle weakness [11]. By assessing postural stability in individuals with bilateral knee OA and healthy controls, we can identify specific impairments that contribute to balance disturbances and functional limitations in the knee OA [12].

The second objective of this study is to assess the correlation between quadriceps strength and postural stability in individuals with bilateral knee OA. Stronger quadriceps muscles can better support the knee joint and maintain proper alignment during weight-bearing activities, thus enhancing overall postural stability [13]. Understanding this correlation is essential as it can inform targeted interventions aimed at improving quadriceps strength to enhance postural stability and reduce the risk of falls in individuals with knee OA [14,15]. The third objective of this study is to assess the mediation effect of pain on the relationship between quadriceps strength and postural stability in individuals with bilateral knee OA. Higher levels of pain may be associated with weaker quadriceps strength and poorer postural stability [16]. This hypothesis is based on previous research suggesting that pain can lead to altered muscle activation patterns and decreased quadriceps strength in knee OA [17]. By elucidating the mediation effect of pain, we can better understand the complex interactions between pain, muscle function, and balance in knee OA, leading to more targeted and effective interventions [18,19]. We hypothesize that individuals with bilateral knee osteoarthritis will exhibit reduced quadriceps strength and impaired postural stability compared to healthy controls. Furthermore, we hypothesize that there is a negative correlation between quadriceps strength and postural stability in knee osteoarthritis individuals. Finally, we hypothesize that pain mediates the relationship between quadriceps strength and postural stability in individuals with bilateral knee osteoarthritis. The findings from this research have the potential to advance our understanding of knee OA pathophysiology and contribute to the development of evidence-based interventions to improve functional outcomes and quality of life for individuals living with this debilitating condition [20,21,22]. Furthermore, the insights gained from this research may pave the way for more effective strategies to manage pain and optimize rehabilitation efforts for better long-term management of bilateral knee OA.

## 2. Materials and Methods

### 2.1. Study Design, Settings, and Ethics

A cross-sectional comparative design was used to assess and compare quadriceps strength, postural stability, and pain mediation in individuals with bilateral knee OA and healthy controls. The study was conducted at medical rehabilitation clinics from June 2021 to March 2023, which included outpatient orthopedic clinics, rehabilitation centers, or community centers with suitable facilities for data collection. Ethical approval was obtained from the University ethics committee (protocol code: REC# 23/22/231 and date of approval: 12 May 2021). All participants provided written informed consent before participating in the study. The study adhered to the principles outlined in the Declaration of Helsinki, ensuring participant confidentiality, privacy, and voluntary participation.

### 2.2. Participants

Participants were recruited through purposive sampling. Knee OA is defined as a degenerative joint disorder characterized by progressive cartilage deterioration, joint pain, stiffness, and functional limitations [23]. The inclusion criteria for the bilateral knee OA group were: (1) Diagnosis of bilateral knee osteoarthritis based on clinical and radiological criteria [24]. (2) Age between 40 and 75 years. (3) Ability to stand and walk independently with or without assistive devices. (4) No recent history of knee surgery or joint replacement. For the healthy control group, the inclusion criteria were: (1) No history of knee joint pain, injury, or any musculoskeletal disorder affecting the lower limbs. (2) Age between 40 and 75 years. (3) Ability to stand and walk independently with no limitations. The inclusion criteria for both groups were based on age and functional mobility, while the exclusion criteria aimed to eliminate confounding factors that could influence quadriceps strength, postural stability, or pain perception. Exclusion criteria for both groups were: (1) Neurological disorders affecting lower limb function. (2) Cardiovascular disorders limiting physical activity. (3) Any other medical condition or history of surgery that may affect the outcome measures.

### 2.3. Quadriceps Strength Assessment

**Figure 1:**
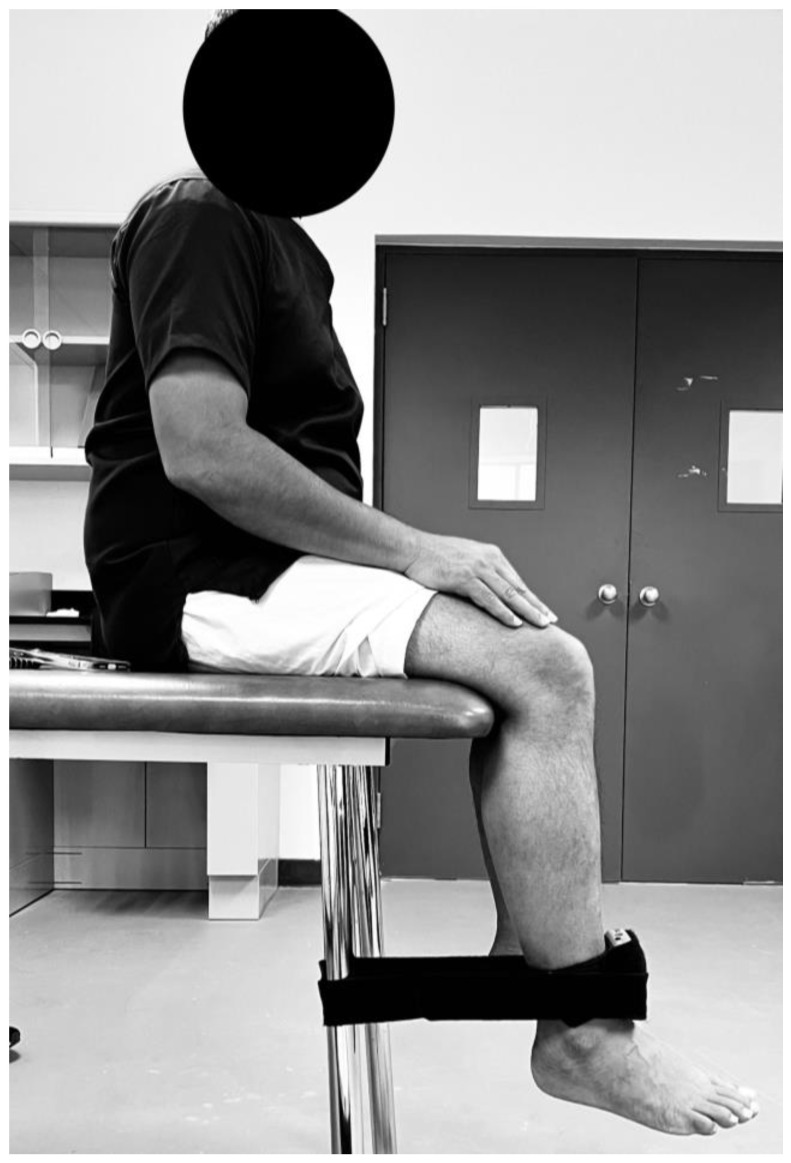
Quadriceps Strength Assessment using a hand-held dynamometer.

The assessment of quadriceps strength involved measuring the maximum voluntary isometric muscle strength. This was achieved using a handheld dynamometer, as illustrated in Figure 1. To conduct this assessment, participants were seated on an examination table, with their knees flexed at a 90° angle and their feet lifted off the ground. To maintain uniformity during the testing process, an inelastic strap was secured around the treatment table underneath the participants. This strap held the handheld dynamometer in place and ensured the constant maintenance of the knee angle throughout the series of trials [25,26]. During the assessment, participants were instructed to extend their knees with maximum effort into the hand-held dynamometer. This exertion was maintained for a duration of 4 s, and the highest force generated during the trial was recorded. To ensure accurate comparisons, three testing trials were conducted for each participant. To account for variations in body mass among individuals, we normalized the average force (measured in Newtons (N)) derived from the three trials by dividing it by the participant’s body mass (measured in N/kg) [27]. This normalization process allowed us to evaluate and compare the quadriceps strength relative to each participant’s weight, thus providing a more standardized measure of muscle strength.

### 2.4. Postural Stability Assessment

**Figure 2:**
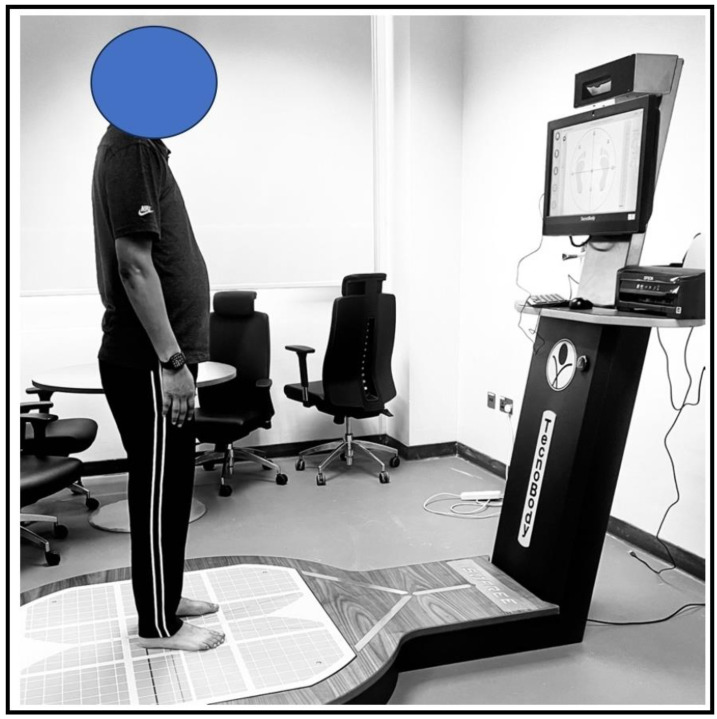
Postural stability assessment in using a stabilometric force platform.

Individuals’ static postural stability was evaluated using a computerized force platform system (Iso-Free). This advanced system incorporates specialized software, a three-dimensional camera, and a monitor that works synchronously to provide real-time postural feedback (see Figure 2). The postural stability assessments were conducted in a quiet and well-ventilated environment to ensure accurate measurements. Before the actual test, participants were given instructions to wear comfortable clothing and relax during the assessment. To familiarize them with the equipment, a practice session was conducted. The examiner carefully calibrated the Iso-Free equipment to ensure accurate data collection. During the postural stability test, each individual was asked to stand barefoot on the force platform in a standardized manner. To maintain balance, participants kept their arms at their sides and focused their gaze on a target mark displayed on the computer monitor (Figure 2). The test required each participant to maintain their balance for a total of 30 s. Three trials were performed for each leg, and the most accurate result from the trials was selected for further analysis. The platform recorded the sway in the anterior-posterior (A/P) and medial–lateral (M/L) directions in millimeters and also measured the area of the sway ellipse in mm^2^, providing comprehensive information about postural stability. To ensure impartiality and unbiased evaluation, a physical therapist administered both the quadriceps strength and postural stability tests without any knowledge of the participants’ characteristics.

Overall, the Iso-Free stabilometric force platform proved to be a valuable tool for assessing static postural stability. Its combination of advanced technology, synchronized components, and real-time feedback allowed for accurate and reliable measurements of individuals’ balance and provided valuable insights for further analysis and intervention planning by healthcare professionals.

### 2.5. Pain Assessment

The patient’s current level of knee pain intensity was assessed using a visual analog scale (VAS) [28]. The VAS is a subjective pain assessment tool commonly used to measure the intensity of pain experienced by individuals [28]. It consists of a horizontal line that ranges from “no pain” at one end to “worst pain imaginable” at the other end, and the line is typically 10 cm in length [28]. Patients are asked to place a mark on the line that represents the level of pain they are currently experiencing, with the distance from the “no pain” end indicating the pain intensity. The VAS allows individuals to quantify their pain on a continuous scale, providing a more precise and sensitive measure of pain intensity compared to other pain assessment methods [28]. The VAS showed excellent reliability and validity values in assessing pain intensity among KOA patients [29].

### 2.6. Sample Size Calculation

The sample size for this study was determined based on a previous study published by Ishii et al. [30], who investigated similar outcome measures and effect sizes in individuals with knee osteoarthritis (OA) and healthy controls. The study by Ishii et al. reported a medium effect size of 0.5 for quadriceps strength and postural stability differences between the knee OA group and asymptomatic individuals. To achieve a statistical power of 80% at a significance level of 0.05, a sample size of 95 participants was calculated for both the knee OA group and the asymptomatic group. Thus, a total sample of 190 participants, including 95 individuals with bilateral knee OA and 95 asymptomatic controls, was recruited for this study.

### 2.7. Data Analysis

**Figure 3:**
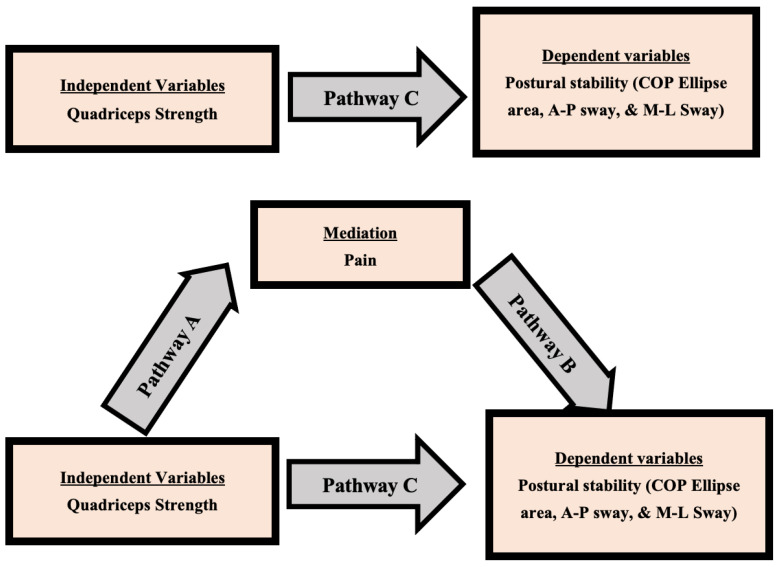
Model of the potential mediating effect of pain on the relationship between quadriceps strength and postural stability variables.

For the analysis of data in this study, SPSS version 24 (IBM Corp., Armonk, NY, USA) was employed. Prior to conducting parametric tests, the normal distribution of the data was evaluated using the Shapiro–Wilk test. The outcomes of the Shapiro–Wilk test revealed that the data related to quadriceps strength, postural stability parameters, and pain scores exhibited a normal distribution (*p* > 0.05). To compare the quadriceps strength and postural stability between the knee OA group and the asymptomatic group, independent samples t-tests were employed. Mean and standard deviation (SD) calculations were performed for each group. The level of statistical significance was set at *p* < 0.05. For the assessment of the relationship between quadriceps strength and postural stability within the knee OA group, Pearson’s correlation coefficient was computed. This coefficient (r) was used to ascertain the strength and direction of the connection between these two variables. To examine the mediation effect of pain on the association between quadriceps strength and postural stability within the knee OA group, a mediation analysis was carried out using a regression-based approach. The central aim of this analysis was to establish whether pain played a significant mediating role in the relationship between quadriceps strength and postural stability, as depicted in Figure 3. The presentation of the data analysis results involved the utilization of appropriate descriptive statistics. Continuous variables were represented using mean and SD values, while categorical variables were expressed as frequencies and percentages.

## 3. Results

The study included 190 participants and the characteristics of the study participants are summarized in Table 1.

The distribution of men and women was comparable between the two groups, with 53 (55.79%) men in both groups. The body mass index (BMI) was higher in the knee OA group (31.3 ± 7 kg/m^2^) compared to the healthy controls group (26.8 ± 4.6 kg/m^2^), resulting in a mean difference of 3.3 kg/m^2^ (95% CI: 1.1–6.0). Pain assessment using the VAS revealed that knee OA participants reported a mean pain score of 5.7 ± 1.9 on a scale of 0–10, while healthy controls reported no pain (0), reflecting the significant impact of pain in the knee OA group.

The Knee injury and Osteoarthritis Outcome Score (KOOS) was used to assess pain, symptoms, and activities of daily living (ADL) function. The knee OA group had significantly lower mean scores in all three domains compared to healthy controls: pain (54.5 ± 13.0 vs. 95.6 ± 13.0), symptoms (56.7 ± 13.8 vs. 93.2 ± 13.7), and ADL function (60.3 ± 13.1 vs. 95.8 ± 6.8). The mean differences were 31.6 (95% CI: 25.5–39.4) for pain, 24.3 (95% CI: 17.6–33.4) for symptoms, and 24.3 (95% CI: 18.4–32.6) for ADL function, indicating significant impairments in knee OA participants. Quality of life, assessed using a numerical scale, demonstrated substantial differences between the two groups. Knee OA participants had a significantly lower quality of life score (33.5 ± 16.6) compared to healthy controls (91.7 ± 12.6), with a mean difference of 44.1 (95% CI: 35.6–55.6), underscoring the considerable impact of knee OA on participants’ overall well-being.

### 3.1. Quadriceps Strength and Postural Stability in Knee OA Participants and Healthy Controls

Table 2 presents the comparisons of quadriceps strength and postural stability measures between knee osteoarthritis (OA) participants (*n* = 95) and healthy controls (*n* = 95), along with the calculated mean differences, 95% confidence intervals (CIs), percentage differences, and effect sizes.

Knee OA = knee osteoarthritis, CI = confidence interval. For quadriceps strength measured in Newtons per kilogram (Nm/kg), both the dominant and non-dominant legs exhibited higher values in healthy controls compared to knee OA participants. The mean difference in quadriceps strength for the dominant leg was 0.18 Nm/kg (95% CI: 0.23–0.37), representing a percentage difference of 14.6% (95% CI: 8.8–23.6%). Similarly, the non-dominant leg showed a mean difference of 0.13 Nm/kg (95% CI: 0.22–0.25), corresponding to a percentage difference of 13.8% (95% CI: 7.6–22.9%). The effect size for the dominant leg was 0.95, while that for the non-dominant leg was 0.89, indicating a substantial effect of knee OA on quadriceps strength. Regarding postural stability, knee OA participants exhibited greater anterior–posterior sway, medial–lateral sway, and ellipse area compared to healthy controls. The mean difference in anterior–posterior sway was 1.96 mm (95% CI: 0.23–1.43), signifying a percentage difference of 22.6% (95% CI: 9.9–31.6%). The medial–lateral sway demonstrated a mean difference of 2.34 mm (95% CI: 1.02–2.54), with a percentage difference of 19.7% (95% CI: 11.6–34.9%). Additionally, the ellipse area showed a mean difference of 235.35 mm^2^ (95% CI: 198.98–412.67), corresponding to a percentage difference of 32.6% (95% CI: 15.8–39.6%). The effect sizes for anterior–posterior sway, medial–lateral sway, and ellipse area were 3.26, 3.89, and 4.95, respectively, signifying significant postural stability impairments in knee OA participants.

### 3.2. Correlation between Quadriceps Strength and Postural Stability

Table 3 illustrates the correlation between quadriceps strength and postural stability variables in individuals with bilateral knee osteoarthritis (OA).

The correlation analysis revealed a significant negative correlation between quadriceps strength in the dominant leg and anterior–posterior sway (r = −0.47, *p* = 0.003), indicating that higher quadriceps strength was associated with reduced anterior–posterior sway. Similarly, the non-dominant leg’s quadriceps strength also exhibited a significant negative correlation with anterior–posterior sway (r = −0.42, *p* = 0.012), emphasizing the association between stronger quadriceps and better anterior–posterior postural control. The results further demonstrated a significant negative correlation between quadriceps strength in both the dominant and non-dominant legs and medial–lateral sway. For the dominant leg, the correlation coefficient (r) was −0.43, with a *p*-value of 0.003. Similarly, the non-dominant leg exhibited a significant negative correlation with medial–lateral sway, with an r-value of −0.41 and a *p*-value of 0.032. Quadriceps strength exhibited a significant negative correlation with the ellipse area, representing overall postural stability. For the dominant leg, the correlation coefficient was −0.51 (*p* < 0.001), indicating that higher quadriceps strength was associated with a smaller ellipse area, reflecting improved postural stability. Similarly, the non-dominant leg’s quadriceps strength demonstrated a significant negative correlation with the ellipse area (r = −0.49, *p* < 0.001), further emphasizing the connection between stronger quadriceps and enhanced postural stability.

### 3.3. Mediation Analysis: Pain as a Mediator

Mediation analysis using pain as a mediator between ankle joint position sense and balance variables is presented in Table 4.

For the interaction between pain, dominant quadriceps strength (QS), and A-P sway, the total effect (c + a × b) was 0.53, with a significant direct effect (c-Path) of 0.24 (*p* < 0.001) and a significant indirect effect (b-Path) of 0.12 (*p* = 0.012). Similarly, for pain, non-dominant QS, and A-P sway interaction, the total effect was 0.52, with a direct effect of 0.31 (*p* < 0.001) and an indirect effect of 0.14 (*p* = 0.014). These results indicate that pain partially mediated the relationship between dominant and non-dominant QS and A-P sway. For pain interacting with both dominant and non-dominant QS and M-L sway, the total effects were 0.53 and 0.49, respectively. The direct effects were 0.29 (*p* < 0.001) and 0.27 (*p* < 0.001), and the indirect effects were 0.16 (*p* = 0.001) and 0.18 (*p* = 0.002). These results indicate that pain partially mediated the relationship between dominant and non-dominant QS and M-L sway. In the interaction between pain, dominant QS, and ellipse area, the total effect was 0.39, with a significant direct effect of 0.26 (*p* < 0.001) and a significant indirect effect of 0.19 (*p* = 0.002). For the interaction between pain, non-dominant QS, and ellipse area, the total effect was 0.48, with a direct effect of 0.21 (*p* < 0.001) and an indirect effect of 0.21 (*p* = 0.002). These results suggest that pain partially mediated the relationship between both dominant and non-dominant QS and ellipse area, reflecting postural stability.

## 4. Discussion

The primary findings of our study reveal significant differences in quadriceps strength and postural stability between individuals with bilateral knee OA and healthy controls. Additionally, we observed significant negative correlations between quadriceps strength and postural stability variables in knee OA participants. Importantly, pain emerged as a significant mediator in the relationship between quadriceps strength and postural stability in individuals with bilateral knee OA.

The observed reduction in quadriceps strength among knee OA participants aligns with well-established literature documenting the association between knee OA and muscle weakness [31,32,33]. Quadriceps muscle atrophy and dysfunction are frequently reported in individuals with knee OA due to factors such as pain-induced disuse, altered neuromuscular activation patterns, and inflammatory processes [34,35]. This muscle weakness is further exacerbated by the pain and biomechanical alterations characteristic of knee OA [36]. Our findings of substantial effect sizes in quadriceps strength differences emphasize the clinical relevance of this observation [37]. The decreased quadriceps strength in knee OA participants can lead to altered joint loading patterns, potentially contributing to disease progression and impaired functional mobility [37].

These findings are consistent with previous studies that have reported reduced quadriceps strength in individuals with knee OA. Yuki et al. [38] found that individuals with knee OA exhibited significantly lower quadriceps strength compared to healthy controls. Similarly, a study by Lim et al. [39] reported a significant decrease in quadriceps strength in knee OA participants compared to controls. Additionally, a study by Hislop et al. [40] found that individuals with unilateral knee osteoarthritis have significantly diminished hip adduction strength (9% lower) and quadriceps strength (16% lower) on the affected side compared to their non-affected side, along with weaker hip abduction, adduction, flexion, and extension strength (with mean differences varying from 16% to 34% lower) in both limbs compared to controls. Additionally, dynamic balance, particularly in posteromedial (4% lower) and anterior (11% lower) directions, was compromised in those with knee osteoarthritis, indicating a bilateral reduction in hip/knee strength and dynamic balance compared to controls [40]. Collectively, these studies, including ours, underline the importance of addressing quadriceps weakness in knee OA management to potentially mitigate functional limitations and enhance overall quality of life.

Our study also revealed compromised postural stability in knee OA participants compared to healthy controls, as indicated by increased anterior–posterior and medial–lateral sway, along with a larger ellipse area [12,41]. These findings align with the altered joint kinematics and proprioceptive deficits often observed in individuals with knee OA. The pain, joint instability, and muscle weakness characteristic of knee OA can disrupt the sensorimotor system’s ability to maintain postural equilibrium [42]. Increased sway and larger ellipse areas suggest challenges in maintaining steady positions and adapting to perturbations, putting individuals at a higher risk of falls [43,44,45,46]. Our findings are consistent with previous research, indicating impaired postural stability in individuals with knee OA. A study by Hassan et al. [47] reported greater postural sway and decreased dynamic balance in knee OA patients compared to healthy controls. Additionally, a study by Wang et al. [48] found that knee OA participants exhibited increased postural sway during quiet stance [48]. Taglietti et al. [49] investigated compromised postural stability in individuals with knee osteoarthritis (OA) in comparison to healthy controls. In this study, women with knee OA exhibited heightened postural sway under eyes open conditions, with statistically significant differences found in the total displacement of sway (TDS; *p* = 0.020), anteroposterior amplitude displacement (APAD; *p* = 0.042), total mean velocity (TMV; *p* = 0.010), and dispersion of the center of pressure (AREA; *p* = 0.045). Intriguingly, Center of Pressure (CoP) variables were unable to differentiate between the two groups, yet a notable negative correlation emerged between postural sway (AREA) and Activities-Specific Balance Confidence Scale (ABC) in closed-eye conditions (rho = −0.42) [49]. Additionally, Hsieh et al. [50] examined postural stability disparities between knee OA patients and controls, revealing lower postural stability scores in knee OA patients (0.7 vs. 0.5, *p* = 0.006), alongside weakened scores in the environmental domain of quality of life (62.2 vs. 66.8, *p* = 0.014). Moreover, knee OA patients displayed weak to moderate associations between postural stability and various International Classification of Functioning, Disability, and Health (ICF) components, including body functions and structures (pain: r = 0.33–0.34, *p* = 0.004; physical fatigue: r = 0.28, *p* = 0.016; reduced motivation: r = 0.30, *p* = 0.011), activities and participation (reduced activity: r = 0.38, *p* = 0.001; physical domain and function: r = 0.34–0.48, *p* = 0.001 to *p* < 0.004; activities of daily living: r = 0.51, *p* < 0.001; sports and recreation: r = 0.35, *p* = 0.003), and personal and environmental factors (age: r = 0.52, *p* < 0.001; quality of life: r = 0.4, *p* = 0.001). [50] These findings underscore the multi-dimensional nature of compromised postural stability in knee OA patients, emphasizing the intricate interplay between physical, functional, and environmental factors. These studies, alongside our results, collectively support the notion that knee OA negatively affects postural stability, emphasizing the need for interventions targeting proprioceptive deficits and muscle weakness to enhance balance and reduce fall risks.

The second objective of our study was to examine the correlation between quadriceps strength and postural stability in individuals with bilateral knee osteoarthritis (OA). This objective aimed to elucidate the potential relationship between muscle strength and the ability to maintain postural stability in the context of knee OA, shedding light on the functional implications of quadriceps weakness [51]. Our results demonstrated significant negative correlations between quadriceps strength and all three measures of postural stability, namely anterior–posterior sway, medial–lateral sway, and ellipse area. These findings suggest that greater quadriceps strength is associated with improved postural stability in individuals with knee OA [52]. The negative correlation implies that as quadriceps strength increases, postural sway and instability decrease, highlighting the integral role of quadriceps strength in maintaining balance and preventing falls [53]. The observed correlations align with the biomechanical role of the quadriceps muscles in stabilizing the knee joint [54]. Quadriceps weakness, frequently seen in knee OA, disrupts the force distribution across the knee joint during weight-bearing activities, potentially leading to altered proprioceptive feedback and compromised postural stability [55]. Weak quadriceps muscles may contribute to difficulties in maintaining a steady stance, especially under perturbing conditions [55]. These findings are in agreement with previous research. A study by Rätsepsoo et al. [56] found a significant association between quadriceps strength and postural stability in individuals with knee OA. Similarly, a study by Hinman et al. [57] reported a relationship between muscle strength and functional performance in individuals with knee OA. These studies, coupled with our results, emphasize that interventions targeting quadriceps strength could potentially enhance postural stability and functional abilities in individuals with knee OA. Addressing quadriceps weakness through targeted exercises, such as resistance training, has shown promise in improving functional outcomes and postural stability [58]. Studies by Ye et al. [52] and Fu et al. [59] demonstrated that quadriceps strengthening exercises improved postural stability and reduced falls in individuals with knee OA. These findings underscore the potential clinical implications of addressing quadriceps weakness as part of comprehensive knee OA management.

The third objective of our study sought to investigate the mediation effect of pain on the relationship between quadriceps strength and postural stability in individuals with bilateral knee OA. This objective aimed to unravel the potential role of pain as a mediator between muscle strength and postural stability, providing insights into the complex interplay between biomechanical and sensory factors in knee OA. Our mediation analysis revealed that pain significantly mediated the relationship between ankle joint position sense, quadriceps strength, and various measures of postural stability. These results suggest that pain may play a role in moderating the impact of quadriceps strength on postural stability [60]. The mediating effect of pain implies that the presence of pain weakens the direct influence of quadriceps strength on postural stability, underscoring the intricate connections between these factors [61]. The observed mediation effect could be attributed to multiple factors. Pain in knee OA may lead to alterations in muscle activation patterns and joint loading strategies, potentially affecting postural stability mechanisms [5]. Pain-induced muscle inhibition can contribute to quadriceps weakness and disrupt the sensory-motor loop involved in maintaining postural stability [62]. Additionally, pain-related fear of movement could lead to protective strategies, altering weight distribution and affecting balance [62]. These combined factors may explain the observed mediation effect, highlighting the multifaceted nature of pain’s influence on postural stability. These findings are in line with previous research. A study by Lai et al. [63] found that pain significantly mediated the relationship between quadriceps strength and physical function in knee OA patients. Additionally, studies by Corrigan et al. [64] and Hicks et al. [65] demonstrated that pain played a mediating role in the association between muscle strength and physical performance. These studies, in conjunction with our results, suggest that addressing pain is crucial not only for pain relief but also for optimizing muscle function and postural stability in individuals with knee OA. The importance of effective pain management in knee OA cannot be overstated. Pain reduction strategies, including pharmacological and non-pharmacological interventions, may have a positive cascading effect on quadriceps strength and subsequently postural stability [66]. Targeting pain modulation alongside interventions to improve quadriceps strength could lead to comprehensive improvements in functional outcomes for individuals with knee OA [66].

### 4.1. Clinical Implications

The findings of this study hold important clinical implications for the management of individuals with bilateral knee OA. The observed deficits in quadriceps strength and compromised postural stability underscore the need for comprehensive interventions that address both muscle weakness and balance impairments. Therapeutic strategies targeting quadriceps strengthening through resistance training and proprioceptive training could potentially mitigate these functional limitations. Furthermore, the mediation effect of pain highlights the critical role of effective pain management strategies in knee OA treatment. Controlling pain not only offers relief but also has the potential to positively impact quadriceps strength and, consequently, postural stability. Clinicians should recognize the interconnected nature of pain perception, muscle function, and postural stability, and tailor interventions to encompass a holistic approach. By addressing both muscle strength and pain, healthcare professionals can enhance functional outcomes, reduce fall risks, and ultimately enhance the overall quality of life for individuals living with bilateral knee OA.

### 4.2. Areas of Future Research

While this study has provided valuable insights into the relationship between quadriceps strength, postural stability, and pain mediation in individuals with bilateral knee OA, future research can further deepen our understanding. Longitudinal studies could unravel the temporal dynamics of these relationships, while mechanistic investigations may elucidate underlying pathways. Exploring intervention strategies targeting pain and quadriceps weakness, incorporating diverse populations, advanced assessment techniques, and neuroimaging methods, could enhance our insights into these complex interactions. By delving into these areas, researchers can develop more targeted interventions to optimize functional outcomes and improve the quality of life for individuals with knee OA.

### 4.3. Limitations of the Study

While this study contributes valuable insights, several limitations must be acknowledged. The cross-sectional design inherently restricts the establishment of causal relationships among variables. The use of convenience sampling might introduce selection bias, potentially limiting the generalizability of the findings to broader populations. The study did not account for potential confounding factors, such as comorbidities or medication use, which could impact the observed relationships. Furthermore, the reliance on self-report measures for pain assessment could introduce subjectivity. Additionally, the study focused on individuals with bilateral knee OA, and extrapolation of these findings to individuals with unilateral OA or other types of knee pathologies should be done cautiously. Finally, while statistical analyses suggest significant associations, the magnitude of these relationships could be influenced by unmeasured variables. Future research addressing these limitations could provide a more comprehensive understanding of the intricate relationships between quadriceps strength, postural stability, pain, and knee OA.

## 5. Conclusions

In summary, our study underscores the importance of addressing quadriceps weakness and postural instability in individuals with bilateral knee osteoarthritis. The significant negative correlations observed between quadriceps strength and postural stability emphasize the need for comprehensive rehabilitation strategies targeting both aspects. Moreover, the mediation effect of pain highlights the intricate relationship among these factors, suggesting potential avenues for more effective management and intervention in knee osteoarthritis.

## Figures and Tables

**Table 1:** Characteristics of the study participants.

Variables	Knee OA Participants (*n* = 95)	Healthy Controls (*n* = 95)	Mean Difference (95% CI)	*p*-Value
Age, years	66.4 ± 7.8 (52 to 80)	66.3 ± 10 (50 to 79)	0.2 (−3, 4)	0.106
Number (%) of men	53 (55.79)	53 (55.79)	-	0.100
Height, cm	167 ± 6.9 (151 to 186)	166.8 ± 7.8 (153 to 181)	2.2 (−4.9, 1.72)	0.236
Body mass, kg	82 ± 16.4 (58 to 117.4)	75.4 ± 15.9 (46.6 to 119.5)	11.3 (3.7, 18.1)	0.016
Body mass index, kg/m^2^	31.3 ± 7 (22.3 to 42.6)	26.8 ± 4.6 (18.1 to 41)	3.3 (1.1, 6.0)	<0.001
Pain VAS (0–10)	5.7 ± 1.9 (2.9 to 8.1)	0	-	-
KOOS (0–100):				
• Pain	54.5 ± 13.0 (21.6 to 76.7)	95.6 ± 13.0 (54.7 to 98)	31.6 (25.5, 39.4)	<0.001
• Symptoms	56.7 ± 13.8 (24 to 83.4)	93.2 ± 13.7 (49 to 99)	24.3 (17.6, 33.4)	<0.001
• ADL function	60.3 ± 13.1 (28.3 to 87.1)	95.8 ± 6.8 (61.2 to 99)	24.3 (18.4, 32.6)	<0.001
Quality of life	33.5 ± 16.6 (0 to 63.4)	91.7 ± 12.6 (36.6 to 100)	44.1 (35.6, 55.6)	<0.001

VAS = visual analog scale; KOOS = Knee injury and Osteoarthritis Outcome Score; ADL = activities of daily living.

**Table 2:** Quadriceps strength and postural stability measures for people with bilateral knee osteoarthritis and healthy participants.

Variables	Knee OA Participants (*n* = 95)	Healthy Controls (*n* = 95)	Mean Difference (95% CI)	Percentage Difference (95% CI)	Effect Size	*p*-Value
Quadriceps strength (Nm/kg)						
• Dominant	1.13 ± 0.54	1.59 ± 0.57	0.18 (0.23, 0.37)	14.6 (8.8, 23.6)	0.95 (0.43, 1.41)	<0.001
• Non-dominant	1.03 ± 0.56	1.48 ± 0.36	0.13 (0.22, 0.25)	13.8 (7.6, 22.9)	0.89 (0.33, 1.40)	<0.001
Postural stability variables						
• Anterior–posterior sway (mm)	9.86 ± 3.017	2.98 ± 1.12	1.96 (0.23, 1.43)	22.6 (9.9, 31.6)	3.26 (1.53, 4.98)	<0.001
• Medial–lateral sway (mm)	7.87 ± 2.23	3.12 ± 1.34	2.34 (1.02, 2.54)	19.7 (11.6, 34.9)	3.89 (1.33, 4.40)	<0.001
• Ellipse area (mm^2^)	935.75 ± 172.56	436.19 ± 135.48	235.35 (198.98, 412.67)	32.6 (15.8, 39.6)	4.95 (2.43, 5.41)	<0.001

**Table 3:** Correlation between quadriceps strength and postural stability variables in bilateral knee osteoarthritis individuals.

Variables		Anterior–Posterior Sway (mm)	Medial–Lateral Sway (mm)	Ellipse Area (mm^2^)
Quadriceps strength (Nm/kg) Dominant	r *p*-value	0.47 0.003	0.43 0.003	0.51 <0.001
Quadriceps strength (Nm/kg) Non-dominant	r *p*-value	0.42 0.012	0.41 0.032	0.49 <0.001

**Table 4:** Mediation analysis using pain as mediation between ankle joint position sense and balance variables.

Test Variables	Total Effect–Direct and Indirect (c + a × b)			Direct Effect (c-Path)			Indirect Effect (b-Path)		
	B	SE	*p*-Value	B	SE	*p*-Value	B	SE	*p*-Value
Pain × Dominant—QS × A-P sway	0.53	0.19	0.023	0.24	0.03	<0.001	0.12	0.04	0.012
Pain × Non-dominant—QS x A-P sway	0.52	0.18	0.019	0.31	0.02	<0.001	0.14	0.05	0.014
Pain × Dominant—QS x M-L sway	0.53	0.17	0.023	0.29	0.03	<0.001	0.16	0.04	0.001
Pain x Non-dominant—QS x M-L sway	0.49	0.16	0.018	0.27	0.02	<0.001	0.18	0.04	0.002
Pain x Dominant—QS x Ellipse area	0.39	0.05	0.021	0.26	0.02	<0.001	0.19	0.05	0.002
Pain x Non-dominant—QS x Ellipse area	0.48	0.08	0.018	0.21	0.02	<0.001	0.21	0.03	0.002

QS, Quadriceps strength; A-P sway, anterior to posterior sway; M-L sway, medial to lateral sway; B, unstandardized coefficients; SE, standard error.

## Data Availability

All data generated or analyzed during this study is available with the corresponding author (RSR) and will be provided on request.

## References

[1] Long H., Liu Q., Yin H., Wang K., Diao N., Zhang Y., Lin J., Guo A. (2022). Prevalence trends of site-specific osteoarthritis from 1990 to 2019: Findings from the Global Burden of Disease Study 2019. Arthritis Rheumatol..

[2] DePhillipo N.N., Aman Z.S., Dekker T.J., Moatshe G., Chahla J., LaPrade R.F. (2021). Preventative and disease-modifying investigations for osteoarthritis management are significantly under-represented in the clinical trial pipeline: A 2020 review. Arthrosc. J. Arthrosc. Relat. Surg..

[3] He Y., Li Z., Alexander P.G., Ocasio-Nieves B.D., Yocum L., Lin H., Tuan R.S. (2020). Pathogenesis of osteoarthritis: Risk factors, regulatory pathways in chondrocytes, and experimental models. Biology.

[4] Chaharmahali L., Gandomi F., Yalfani A., Fazaeli A. (2021). The effect of self-reported knee instability on plantar pressure and postural sways in women with knee osteoarthritis. J. Orthop. Surg. Res..

[5] Zeng Z., Shan J., Zhang Y., Wang Y., Li C., Li J., Chen W., Ye Z., Ye X., Chen Z. (2022). Asymmetries and relationships between muscle strength, proprioception, biomechanics, and postural stability in patients with unilateral knee osteoarthritis. Front. Bioeng. Biotechnol..

[6] Coaccioli S., Sarzi-Puttini P., Zis P., Rinonapoli G., Varrassi G. (2022). Osteoarthritis: New insight on its pathophysiology. J. Clin. Med..

[7] Whittaker J., Runhaar J., Bierma-Zeinstra S., Roos E. (2021). A lifespan approach to osteoarthritis prevention. Osteoarthr. Cartil..

[8] Shorter E., Sannicandro A.J., Poulet B., Goljanek-Whysall K. (2019). Skeletal muscle wasting and its relationship with osteoarthritis: A mini-review of mechanisms and current interventions. Curr. Rheumatol. Rep..

[9] Burgess L.C., Taylor P., Wainwright T.W., Bahadori S., Swain I.D. (2021). Adherence to neuromuscular electrical stimulation interventions for muscle impairment in hip and knee osteoarthritis: A systematic review. Clin. Med. Insights Arthritis Musculoskelet. Disord..

[10] Promsri A., Haid T., Werner I., Federolf P. (2020). Leg dominance effects on postural control when performing challenging balance exercises. Brain Sci..

[11] Zhang P., Liu F., He X., Brooke-Wavell K., Song Q., Fong D.T. (2023). Effect of biophysical interventions on balance and postural control in patients with ankle instability: A systematic review. Med. Nov. Technol. Devices.

[12] Raizah A., Reddy R.S., Alshahrani M.S., Tedla J.S., Dixit S., Gular K., Gautam A.P., Ahmad I., Kandakurti P.K. (2023). Investigating Knee Joint Proprioception and Its Impact on Limits of Stability Using Dynamic Posturography in Individuals with Bilateral Knee Osteoarthritis—A Cross-Sectional Study of Comparisons and Correlations. J. Clin. Med..

[13] Truszczyńska-Baszak A., Dadura E., Drzał-Grabiec J., Tarnowski A. (2020). Static balance assessment in patients with severe osteoarthritis of the knee. Knee.

[14] ALMohiza M.A., Reddy R.S., Alkhamis B.A., Alghamdi N.H., Alshahrani A., Ponneru B.R., Mukherjee D. (2023). A Cross-Sectional Study Investigating Lumbar Proprioception Impairments in Individuals with Type 2 Diabetes Mellitus: Correlations with Glycated Hemoglobin Levels. Biomedicines.

[15] Reddy R.S., Tedla J.S., Dixit S., Raizah A., Al-Otaibi M.L., Gular K., Ahmad I., Sirajudeen M.S. (2022). Cervical joint position sense and its correlations with postural stability in subjects with fibromyalgia syndrome. Life.

[16] Stief F., Sohn A., Vogt L., Meurer A., Kirchner M. (2023). Characterization of Postural Sway in Women with Osteoporosis and a Control Group by Means of Linear and Nonlinear Methods. Bioengineering.

[17] Hatefi M., Hadadnezhad M., Shojaedin S., Babakhani F., Tazji M.K. (2023). The effects of the Posterior X Taping versus augmented feedback on lower-extremity kinematic and muscle activity pattern during unilateral weight-bearing activities in men with tibiofemoral varus malalignment. J. Exp. Orthop..

[18] Alfaya F.F., Reddy R.S., Alshahrani M.S., Tedla J.S., Dixit S., Gular K., Mukherjee D. (2023). Investigating the Mediating Role of Pain in the Relationship between Ankle Joint Position Sense and Balance Assessed Using Computerized Posturography in Individuals with Unilateral Chronic Ankle Instability: A Cross-Sectional Study. Appl. Sci..

[19] Zhen G., Fu Y., Zhang C., Ford N.C., Wu X., Wu Q., Yan D., Chen X., Cao X., Guan Y. (2022). Mechanisms of bone pain: Progress in research from bench to bedside. Bone Res..

[20] Buck A.N., Vincent H.K., Newman C.B., Batsis J.A., Abbate L.M., Huffman K.F., Bodley J., Vos N., Callahan L.F., Shultz S.P. (2023). Evidence-Based Dietary Practices to Improve Osteoarthritis Symptoms: An Umbrella Review. Nutrients.

[21] Berteau J.-P. (2022). Knee Pain from Osteoarthritis: Pathogenesis, Risk Factors, and Recent Evidence on Physical Therapy Interventions. J. Clin. Med..

[22] Alahmari K.A., Kakaraparthi V.N., Reddy R.S., Silvian P., Tedla J.S., Rengaramanujam K., Ahmad I. (2021). Combined effects of strengthening and proprioceptive training on stability, balance, and proprioception among subjects with chronic ankle instability in different age groups: Evaluation of clinical outcome measures. Indian J. Orthop..

[23] Stoddart J.C., Dandridge O., Garner A., Cobb J., van Arkel R.J. (2021). The compartmental distribution of knee osteoarthritis–a systematic review and meta-analysis. Osteoarthr. Cartil..

[24] Runhaar J., Kloppenburg M., Boers M., Bijlsma J., Bierma-Zeinstra S., Group C.E. (2021). Towards developing diagnostic criteria for early knee osteoarthritis: Data from the CHECK study. Rheumatology.

[25] Schaubert K.L., Bohannon R.W. (2005). Reliability and validity of three strength measures obtained from community-dwelling elderly persons. J. Strength Cond. Res..

[26] Mentiplay B.F., Perraton L.G., Bower K.J., Adair B., Pua Y.-H., Williams G.P., McGaw R., Clark R.A. (2015). Assessment of lower limb muscle strength and power using hand-held and fixed dynamometry: A reliability and validity study. PLoS ONE.

[27] Culvenor A.G., Wirth W., Ruhdorfer A., Eckstein F. (2016). Thigh muscle strength predicts knee replacement risk independent of radiographic disease and pain in women: Data from the osteoarthritis initiative. Arthritis Rheumatol..

[28] Begum M.R., Hossain M.A. (2019). Validity and reliability of visual analogue scale (VAS) for pain measurement. J. Med. Case Rep. Rev..

[29] Agun M., Adedoyin R.A., Anifaloba R. (2003). Reliability and concurrent validity of visual analogue scale and modified verbal rating scale of pain assessment in adult patients with knee osteoarthritis in Nigeria. S. Afr. J. Physiother..

[30] Ishii Y., Noguchi H., Sato J., Ishii H., Ishii R., Toyabe S.-I. (2020). Association of knee osteoarthritis grade with one-leg standing balance and quadriceps strength in male independent ambulators aged ≥ 80 years. J. Orthop..

[31] Chang A.H., Chmiel J.S., Almagor O., Hayes K.W., Guermazi A., Prasad P.V., Moisio K.C., Zhang Y., Szymaszek J., Sharma L. (2019). Hip muscle strength and protection against structural worsening and poor function and disability outcomes in knee osteoarthritis. Osteoarthr. Cartil..

[32] Marks R. (2016). Muscle Function during Selected Weight-Bearing Activities in Adults with Knee Osteoarthritis-A Narrative Overview. Int. J. Orthop..

[33] Pierce B. (2009). The Severity of Obesity and Knee Osteoarthritis: Effects on Strength and Gait. Ph.D. Thesis.

[34] Dalle S., Koppo K. (2020). Is inflammatory signaling involved in disease-related muscle wasting? Evidence from osteoarthritis, chronic obstructive pulmonary disease and type II diabetes. Exp. Gerontol..

[35] Alahmari K.A., Reddy R.S., Silvian P., Ahmad I., Kakarparthi V.N., Rengaramanujam K. (2019). Intra and inter-rater reliability for deep neck flexor and neck extensor muscle endurance tests in subjects with and without subclinical neck pain. Phys. Med. Rehabil. Kurortmed..

[36] Miller M.S., Callahan D.M., Toth M.J. (2014). Skeletal muscle myofilament adaptations to aging, disease, and disuse and their effects on whole muscle performance in older adult humans. Front. Physiol..

[37] Tayfur B., Charuphongsa C., Morrissey D., Miller S.C. (2021). Neuromuscular function of the knee joint following knee injuries: Does it ever get back to normal? A systematic review with meta-analyses. Sports Med..

[38] Ito Y., Aoki T., Sato T., Oishi K., Ishii K. (2020). Comparison of quadriceps setting strength and knee extension strength tests to evaluate lower limb muscle strength based on health-related physical fitness values in elderly people. BMJ Open Sport Exerc. Med..

[39] Lim B.W., Hinman R.S., Wrigley T.V., Sharma L., Bennell K.L. (2008). Does knee malalignment mediate the effects of quadriceps strengthening on knee adduction moment, pain, and function in medial knee osteoarthritis? A randomized controlled trial. Arthritis Care Res. Off. J. Am. Coll. Rheumatol..

[40] Hislop A., Collins N.J., Tucker K., Semciw A.I. (2022). Hip strength, quadriceps strength and dynamic balance are lower in people with unilateral knee osteoarthritis compared to their non-affected limb and asymptomatic controls. Braz. J. Phys. Ther..

[41] Suda E.Y., Hirata R.P., Palsson T., Vuillerme N., Sacco I.C., Graven-Nielsen T. (2019). Experimental knee-related pain enhances attentional interference on postural control. Eur. J. Appl. Physiol..

[42] Hurley M.V., Scott D.L., Rees J., Newham D.J. (1997). Sensorimotor changes and functional performance in patients with knee osteoarthritis. Ann. Rheum. Dis..

[43] Alshahrani M.S., Reddy R.S. (2022). Relationship between Kinesiophobia and ankle joint position sense and postural control in individuals with chronic ankle instability—A cross-sectional study. Int. J. Environ. Res. Public Health.

[44] Asiri F., Reddy R.S., Alshahrani M.S., Tedla J.S., Dixit S., Alshahrani A., Gular K., Raizah A. (2023). Mediation Effect of Pain on the Relationship between Kinesiophobia and Postural Control: Comparison and Correlations in Individuals with Fibromyalgia Syndrome and Asymptomatic Individuals—A Cross-Sectional Study. Life.

[45] Kandakurti P.K., Reddy R.S., Kakarparthy V.N., Rengaramanujam K., Tedla J.S., Dixit S., Gautam A.P., Silvian P., Gular K., Eapen C. (2021). Comparison and association of neck extensor muscles’ endurance and postural function in subjects with and without chronic neck pain–a cross-sectional study. Phys. Med. Rehabil. Kurortmed..

[46] Neelapala Y.R., Reddy Y.R.S., Danait R. (2016). Effect of mulligan’s posterolateral glide on shoulder rotator strength, scapular upward rotation in shoulder pain subjects–a randomized controlled trial. J. Musculoskelet. Res..

[47] Hassan B.S., Mockett S., Doherty M. (2001). Static postural sway, proprioception, and maximal voluntary quadriceps contraction in patients with knee osteoarthritis and normal control subjects. Ann. Rheum. Dis..

[48] Wang J., Severin A.C., Mears S.C., Stambough J.B., Barnes C.L., Mannen E.M. (2021). Changes in Mediolateral Postural Control Mechanisms During Gait After Total Knee Arthroplasty. J. Arthroplast..

[49] Taglietti M., Bela L.F.D., Dias J.M., Pelegrinelli A.R.M., Nogueira J.F., Júnior J.P.B., da Silva Carvalho R.G., McVeigh J.G., Facci L.M., Moura F.A. (2017). Postural sway, balance confidence, and fear of falling in women with knee osteoarthritis in comparison to matched controls. PMR.

[50] Hsieh R.-L., Lee W.-C., Lo M.-T., Liao W.-C. (2013). Postural stability in patients with knee osteoarthritis: Comparison with controls and evaluation of relationships between postural stability scores and international classification of functioning, disability and health components. Arch. Phys. Med. Rehabil..

[51] Nieto-Guisado A., Solana-Tramunt M., Marco-Ahulló A., Sevilla-Sánchez M., Cabrejas C., Campos-Rius J., Morales J. (2022). The mediating role of vision in the relationship between proprioception and postural control in older adults, as compared to teenagers and younger and middle-aged adults. Healthcare.

[52] Ye J., Simpson M.W., Liu Y., Lin W., Zhong W., Cai S., Zou L. (2020). The effects of baduanjin qigong on postural stability, proprioception, and symptoms of patients with knee osteoarthritis: A randomized controlled trial. Front. Med..

[53] Jeon W., Whitall J., Alissa N., Westlake K. (2022). Age-related differences in stepping stability following a sudden gait perturbation are associated with lower limb eccentric control of the perturbed limb. Exp. Gerontol..

[54] Rubin D.A., Rose D.J., Escano D.L., Holmes S.C., Garcia S.A., Pamukoff D.N. (2023). Contributing factors to postural stability in Prader-Willi syndrome. Hum. Mov. Sci..

[55] Efstathiou M.A., Giannaki C.D., Roupa Z., Hadjisavvas S., Stefanakis M. (2022). Evidence of distorted proprioception and postural control in studies of experimentally induced pain: A critical review of the literature. Scand. J. Pain.

[56] Rätsepsoo M., Gapeyeva H., Vahtrik D., Aibast H., Ereline J., Haviko T., Märtson A., Pääsuke M. (2011). Knee pain and postural stability in women with gonarthrosis before and six months after unilateral total knee replacement. Acta Kinesiol. Univ. Tartu..

[57] Holden M.A., Hattle M., Runhaar J., Riley R.D., Healey E.L., Quicke J., van der Windt D.A., Dziedzic K., van Middelkoop M., Burke D. (2023). Moderators of the effect of therapeutic exercise for knee and hip osteoarthritis: A systematic review and individual participant data meta-analysis. Lancet Rheumatol..

[58] Black W.R., DiCesare C.A., Thomas S., Pfeiffer M., Williams S.E., Kitchen K., Ting T.V., Myer G.D., Kashikar-Zuck S. (2021). Preliminary evidence for the Fibromyalgia Integrative Training Program (FIT Teens) improving strength and movement biomechanics in juvenile fibromyalgia: Secondary analysis and results from a pilot randomized clinical trial. Clin. J. Pain.

[59] Fu S., Duan T., Hou M., Yang F., Chai Y., Chen Y., Liu B., Ma Y., Liu A., Wang X. (2021). Postural balance in individuals with knee osteoarthritis during stand-to-sit task. Front. Hum. Neurosci..

[60] Ferrer-Peña R., Cuenca-Martínez F., Romero-Palau M., Flores-Román L.M., Arce-Vázquez P., Varangot-Reille C., Suso-Martí L. (2021). Effects of motor imagery on strength, range of motion, physical function, and pain intensity in patients with total knee arthroplasty: A systematic review and meta-analysis. Braz. J. Phys. Ther..

[61] Munsch A.E. (2023). Optimizing Knee Joint Loading: Association Between Quadriceps Contractile Behavior, Knee Joint Biomechanics, and Cartilage Contact Forces. Ph.D. Thesis.

[62] Rahman-Enyart M. (2022). The Efficacy of Perturbation-Based Balance Training on Preventing Falls in Older Adults: A Systematic Review and Synthesis without Meta-Analysis (SWiM). Ph.D. Thesis.

[63] Lai Z., Lee S., Chen Y., Wang L. (2021). Comparison of whole-body vibration training and quadriceps strength training on physical function and neuromuscular function of individuals with knee osteoarthritis: A randomised clinical trial. J. Exerc. Sci. Fit..

[64] Corrigan P., Neogi T., Frey-Law L., Jafarzadeh S., Segal N., Nevitt M., Lewis C., Stefanik J. (2023). Relation of pain sensitization to self-reported and performance-based measures of physical functioning: The Multicenter Osteoarthritis (MOST) study. Osteoarthr. Cartil..

[65] Hicks C., Levinger P., Menant J.C., Lord S.R., Sachdev P.S., Brodaty H., Sturnieks D.L. (2020). Reduced strength, poor balance and concern about falls mediate the relationship between knee pain and fall risk in older people. BMC Geriatr..

[66] Shahid A., Inam-Ur-Raheem M., Nawaz M.Y., Rashid M.H., Oz F., Proestos C., Aadil R.M. (2022). Diet and lifestyle modifications: An update on non-pharmacological approach in the management of osteoarthritis. J. Food Process. Preserv..

